# Myeloperoxidase induces monocyte migration and activation after acute myocardial infarction

**DOI:** 10.3389/fimmu.2024.1360700

**Published:** 2024-04-26

**Authors:** Vera B.M. Peters, Friederike Matheis, Immanuel Erdmann, Harshal N. Nemade, David Muders, Martin Toubartz, Merve Torun, Dennis Mehrkens, Simon Geißen, Felix Sebastian Nettersheim, Felix Picard, Henning Guthoff, Alexander Hof, Per Arkenberg, Birgit Arand, Anna Klinke, Volker Rudolph, Hinrich Peter Hansen, Daniel Bachurski, Matti Adam, Friedrich Felix Hoyer, Holger Winkels, Stephan Baldus, Martin Mollenhauer

**Affiliations:** ^1^ Heart Center, Department of Cardiology, Faculty of Medicine and University Hospital Cologne, University of Cologne, Cologne, Germany; ^2^ Center for Molecular Medicine Cologne, University of Cologne, Cologne, Germany; ^3^ Clinic for General and Interventional Cardiology/Angiology, Agnes Wittenborg Institute for Translational Cardiovascular Research, Herz- und Diabeteszentrum Nordrhein Westfalen (NRW), University Hospital of the Ruhr-Universität Bochum, Bad Oeynhausen, Germany; ^4^ Cluster of Excellence on Cellular Stress Responses in Aging-Associated Diseases (CECAD), University of Cologne, Cologne, Germany; ^5^ Department I of Internal Medicine, Center for Integrated Oncology Aachen Bonn Cologne Duesseldorf, Faculty of Medicine and University Hospital Cologne, University of Cologne, Cologne, Germany

**Keywords:** heart failure, myeloperoxidase, monocytes, myocardial infarction, migration

## Abstract

**Introduction:**

Myocardial infarction (MI) is a significant contributor to morbidity and mortality worldwide. Many individuals who survive the acute event continue to experience heart failure (HF), with inflammatory and healing processes post-MI playing a pivotal role. Polymorphonuclear neutrophils (PMN) and monocytes infiltrate the infarcted area, where PMN release high amounts of the heme enzyme myeloperoxidase (MPO). MPO has numerous inflammatory properties and MPO plasma levels are correlated with prognosis and severity of MI. While studies have focused on MPO inhibition and controlling PMN infiltration into the infarcted tissue, less is known on MPO’s role in monocyte function.

**Methods and results:**

Here, we combined human data with mouse and cell studies to examine the role of MPO on monocyte activation and migration. We revealed a correlation between plasma MPO levels and monocyte activation in a patient study. Using a mouse model of MI, we demonstrated that MPO deficiency led to an increase in splenic monocytes and a decrease in cardiac monocytes compared to wildtype mice (WT). In vitro studies further showed that MPO induces monocyte migration, with upregulation of the chemokine receptor CCR2 and upregulation of inflammatory pathways identified as underlying mechanisms.

**Conclusion:**

Taken together, we identify MPO as a pro-inflammatory mediator of splenic monocyte recruitment and activation post-MI and provide mechanistic insight for novel therapeutic strategies after ischemic injury.

## 1 Introduction

Cardiovascular disease (CVD) remains the leading cause of death worldwide, with ischemic heart disease, including myocardial infarction (MI), causing 16.17% of all global deaths over the last three decades (1). Treatment of acute MI involves early reperfusion therapies followed by chronic use of medication. Although advances in treatment have improved survival after MI (2), many patients surviving the acute event continue to develop heart failure (HF) (3, 4). HF affects more than 64 million people worldwide (5) and is a major economic burden (6). Its development after MI involves a complex inflammatory cascade subsequent to cardiac tissue damage (7).

Post-MI healing with subsequent HF development is predominantly considered a three-step process: induction of inflammation, resolution of inflammation, and ventricular scar formation (8). Acute MI triggers the release of damage-associated molecular patterns (DAMPs) by dead and dying cardiomyocytes. Together with the release of cytokines, growth factors, and chemokines by activated resident macrophages and fibroblasts, this causes a massive infiltration of polymorphonuclear neutrophils (PMN) and monocytes into the area of infarction. Cardiac PMN infiltration surges within the initial 6 h, reaching its peak at 24 h (9). Circulating proinflammatory Ly6C^high^ monocytes are recruited from splenic reservoirs and later on from the bone marrow in a CCR2-dependent manner (10–12). Splenic monocytes accumulate in the injured myocardial tissue within 24 h post-MI (13), expand rapidly, and give rise to inflammatory and reparative macrophage populations (14).

Infiltrating PMN release high amounts of the oxidative enzyme myeloperoxidase (MPO). MPO is a heme enzyme that catalyzes the production of highly reactive hypohalous acids such as hypochlorous acid (HOCl), thereby reducing its principal substrate, hydrogen peroxide (H_2_O_2_). Although inflammation is crucial for cardiac healing post-MI, excessive inflammation induces high release of MPO by PMN and results in adverse cardiac remodeling and an increased risk of secondary events (15). Furthermore, MPO plasma levels are associated with prognosis and severity of MI (16) and are an important biomarker for identifying patients at risk (17). Moreover, it showed numerous properties relevant to cardiovascular disease, e.g., affecting apoptosis, PMN migration and activation, NETosis, platelet function, adaptive immunity, endothelial dysfunction, lipoprotein modification, vascular function, and leukocyte recruitment, as reviewed in refs (18–20).

Controlling neutrophil infiltration could offer another way to reduce excess inflammation post-MI and subsequent HF. Overactivation of PMN results in increased cardiac damage (21), whereas complete depletion of PMN results in uncontrolled myocardial fibrosis and declined cardiac function post-MI (22). Also, cardiac macrophage depletion post-MI resulted in impaired healing, meaning more sensitive mechanisms need to be discovered to modulate the initial inflammatory leukocyte response to increase cardiac healing post-MI (23). Hence, research has focused on pharmacological inhibition of MPO, which improved ventricular function and tissue remodeling in long-term experimental MI mouse models (17, 24, 25). The MPO inhibitor AZD4831 is currently subject to (pre) clinical trials (26, 27).

Therapeutically targeting MPO may modify the inflammatory cascade and repair it during the acute phase of MI. While studies have focused on MPO inhibition and controlling PMN infiltration into the infarcted tissue, less is known on MPO’s role in monocyte migration and activation in MI. Here, we identify MPO as a proinflammatory mediator of splenic monocyte recruitment and activation after MI and provide mechanistic insight for potential therapeutic targets after ischemic injury.

## 2 Methods

### 2.1 Patient inclusion

Patients of the cardiology ward of the Heart Center of University Hospital Cologne with an ejection fraction (EF) of < 45% as measured by transthoracic echocardiography were included. Severe infection or severe cardiac decompensation were considered exclusion criteria. The control group consisted of age-matched, nonhospitalized volunteers without known heart disease. A total of 42 patients and 21 controls were included in the study. Consent was obtained, and blood collection was approved by ethics application number 13-019.

### 2.2 Group characteristics

A total of 42 patients and 21 controls were included in the study. Group characteristics can be found in **Supplementary Table S1**. There was no difference in age, gender, diagnosed hypertension, hypercholesterolemia, diabetes, or smoking status. None of the control subjects suffered from kidney failure. Ischemic cardiomyopathy was the causative factor for HF in 59.5% of the patients. Correlation analyses of MPO plasma levels and TNF-α expression by monocytes were made, randomly including 17 patients and seven controls.

### 2.3 Human monocyte isolation

Blood was drawn from the cubital vein. Plasma was stored at −80°C, and monocytes were isolated within 2–4 h after collection. Heparinized blood was loaded into SepMate falcons (STEMCELL Technologies, Vancouver, Canada) containing Lympocyte Separation Medium (PromoCell, Heidelberg, Germany). PBMCs were washed and incubated with Monocyte Attachment Medium (PromoCell) for 75 min at 37°C, 5% CO_2_. Nonadherent cells and monocytes were incubated for another 2 h prior to RNA isolation. Culture media was stored at −80°C for further analyses.

### 2.4 RNA isolation

RNA was isolated according to manufacturer’s protocol using RNeasy Mini Kit (Qiagen, Hilden, Germany) and quantified using Nano Drops 200 (Thermo Fisher Scientific, Waltham, MA, USA). RNA was stored at −80°C.

### 2.5 Reverse transcription

mRNA was reverse transcribed to cDNA using a High Capacity cDNA Reverse Transcription Kit (Thermo Fisher Scientific) according to manufacturer’s protocol.

### 2.6 Primer design

All required mRNA sequences were obtained from the PubMed nucleotide database. Primer3Plus and Nucleotide Blast were used for primer design. Primers were ordered from Qiagen. All used primers can be found in **Supplementary Table S2**.

### 2.7 Quantitative real-time PCR

Quantitative real-time PCR analyses were performed according to manufacturer’s protocol (Bio-Rad, Hercules, CA, USA). The CFX96 Touch Real-Time PCR Detection System (Bio-Rad) was used for qRT analysis. Each sample was normalized against a reference gene.

### 2.8 ELISA

Cells were lysed and samples were purified from cell residues and analyzed for MPO (Hycult Biotech, Uden, the Netherlands) and TNF-α (R&D Systems, Minneapolis, MN) using ELISA according to manufacturer’s protocol.

### 2.9 Animal studies and ethics statement

All animal studies were conducted using 8- to 14-week-old *Mpo^−/−^* and wild-type (WT) mice with C57BL/6J background (The Jackson Laboratory, Bar Harbor, ME, USA). The strategy for generation of the *Mpo^−/−^* mice has been previously described (28). All animal studies were approved by the local authorities (State Agency for Nature, Environment and Consumer Protection (LANUV), Recklinghausen, NRW, Germany) and under license numbers 2014.A234 and 2020.A487. All surgical interventions were performed under anesthesia using isoflurane and perioperative analgesia with buprenorphine.

### 2.10 Injection model

*Mpo^−/−^* mice received an i.p. injection with 0.9% NaCl, 10 µg/ml MPO in 0.9% NaCl or 3% thioglycolate medium in 0.9% NaCl. Mice were killed after 3 days, and peritoneal cells were counted (Bio-Rad TC20 Automated Cell Counter). Viability was checked using trypan blue, and counting gates were set to 7–40 µm to exclude erythrocytes and debris.

### 2.11 Left anterior descending artery ligation

Mice were anesthetized with isoflurane, received a subcutaneous injection of 0.05 mg/kg bodyweight buprenorphine (Essex-Pharma, Munich, Germany), and were placed on a heating pad to regulate body temperature. After endotracheal intubation, animals were ventilated with 150 strokes/min and a stroke volume of 7 µL/g bodyweight (Harvard Apparatus, Holliston, MA, USA). Permanent ligation of the left anterior descending artery (LAD) was performed using a dissecting microscope (Leica MZ6, Leica Microsystems, Wetzlar, Germany). After lateral thoracotomy of the fourth intercostal space, an 8/0 polypropylene suture (Polypro, CP Medical, Norcross, GA, USA) was placed around the LAD, and the artery was ligated with a bow tie. Ischemia was visually confirmed by blanching of the left ventricular (LV) apex.

Mice were killed on days 1 or 3 after ligation and received a subcutaneous injection of buprenorphine (0.05 mg/kg) twice a day.

Animals that died during instrumentations, or that did not properly recover, were excluded from analyses.

While previous work from our lab never showed any major effects in sham animals (17, 25), we only included mice undergoing LAD surgery in our analyses and used wild-type mice as a control for *Mpo^−/−^* mice.

### 2.12 Collection of blood

Blood was drawn under deep isoflurane anesthesia by heart puncture. Blood counts were taken using a Veterinary Hematology Analyzer (Heska Element HT5, Heska, Loveland, CO, USA), and plasma was stored at −80°C.

### 2.13 Immunofluorescent staining

Hearts and spleens were collected and stored at −80°C until they were cut for staining purposes. Antibody details can be found in **Supplementary Table S3**. Frozen heart and spleen sections (8 µm thickness) in OCT compound (Sakura Finetek, Alphen aan den Rijn, the Netherlands) were fixed with acetone. Sections were incubated with an Fc-block CD16/CD32 solution (BD, Franklin Lakes, NJ, USA). Tissue sections were stained with antibodies to Ly6G (1:200 (BioLegend, San Diego, CA, USA) or CD11b (1:100, Invitrogen, Waltham, MA, USA) for 1 h. Nuclei were marked by DAPI staining. DAKO Fluorescence mounting medium (Agilent Technologies, Santa Clara, CA, USA) was used for mounting the slides. The tissue was fixed overnight in 4% paraformaldehyde (PFA) at 4°C. Images were acquired (Keyence microscope BZ-X800 Series, Keyence, Osaka, Japan) in five sections per organ and staining and analyzed using ImageJ.

### 2.14 Cardiac cell isolation

After blood withdrawal, the heart was flushed, and the left ventricle (LV), including the septum was collected. Tissue weight was taken before being cut into small pieces. LVs were digested in an enzymatic solution (a mixture of collagenase I, collagenase XI, hyaluronase, and DNase I in HBSS) and filtered. Cells were recovered in warm RPMI + 10% FBS before being resuspended in FACS buffer until further processing.

### 2.15 Splenocyte isolation

The spleen was collected, and tissue weight was taken. The spleen was put through a 70-µm cell strainer and resuspended in FACS buffer. Cell suspension was filtered through a 50-µm cell strainer, and erythrocytes were lysed using RBC lysis buffer. Cells were resuspended in the FACS buffer until further processing.

### 2.16 Flow cytometry staining procedure

Isolated cells from LVs and spleens were added to 96-well plates for staining of neutrophils and monocytes. All antibodies were diluted in FACS buffer, and antibody details can be found in **Supplementary Table S4**. Cells were incubated with FcR block (anti-CD16/32, 1:100, BD) and Live Dead UV (1:1,000, BioLegend). After washing, mastermix for extracellular staining was added. Cells were fixed using 2% PFA in FACS buffer. Cells were diluted in FACS buffer and kept at 4°C until acquisition on a Cytek Aurora flow cytometer (Cytek, Fremont, CA, USA). Analyses were performed with FlowJo software (Ashland, OR, USA). Neutrophils were defined as live CD45^+^CD3e^-^Ly6G^+^ cells. Monocytes were defined as live CD45^+^CD3e^−^Ly6G^−^CD19^−^CD11b^+^CD115^+^Ly6C^hi^ cells (gating strategy shown in **Supplementary Figure S1**).

### 2.17 Cell culture

THP-1 monocytic cells were cultured in RPMI glutaMAX medium supplemented with 10% FBS and 1% penicillin/streptomycin (all Thermo Fisher Scientific) and split twice a week.

### 2.18 Activation of THP-1 cells by MPO

Cells were washed with PBS, counted using a cell counter (Bio-Rad TC20 Automated Cell Counter), and incubated in equal numbers with either plain cell culture medium, 10 ng/ml MPO (Planta Natural Products, Vienna, Austria) or 10 ng/ml MPO plus 0.04 µM H_2_O_2_. Cells were incubated for 24 h at 37°C, 5% CO_2_, and collected for migration assay or RNA isolation.

### 2.19 Migration assay

Cells were added onto a precoated chemotaxis slide (u-slide Luer, ibidi, Gräfelfing, Germany) according to manufacturer’s protocol. MCP-1 (100 ng/ml, Thermo Fisher Scientific) was added as a chemoattractant, and cell migration was measured over the following 5 h at 37°C and 5% CO_2_ using live-cell imaging with temperature and CO_2_ regulation (Keyence BY-X800E Compact Fluorescence Microscope). Cell migration was analyzed by following 10 cells per slide using TrackMate software with manual tracking.

### 2.20 Western blot

Incubated THP-1 cells were lysed in RIPA buffer, and the protein amount was analyzed using a BCA assay kit (Thermo Fisher Scientific). Samples were diluted to equal concentrations of protein using Laemmli buffer and denatured at 95°C. The gel (Bio-Rad) was loaded in duplicates and ran for 1 h at 100 V before it was blotted to a membrane (Amersham, Cytiva, Marlborough, MA, USA) by Mini-Protean Tetra System (Bio-Rad). Ponceau staining (SERVA, Heidelberg, Germany) was performed to quantify the loaded proteome. Blots were blocked with a 3% BSA-TBST solution, and staining was performed by the Protein Detection System (Merck Millipore, Burlington, MA, USA).

The gel was incubated with antibodies to pNF-κB (Invitrogen) and pMAP-K (Cell Signaling Technology, Danvers, MA, USA), followed by a secondary anti-rabbit antibody (Vector Laboratories, Newark, CA, USA). Images were acquired by Fusion FX7 (Peqlab Biotechnologie, Erlangen, Germany) with an ECL Western Blotting Detection Reagent (Cytiva). Stripping with NaOH was performed before the blocking and staining procedure was repeated with unphosphorylated primary antibodies to total MAP-K p38 (Thermo Fisher Scientific) and NF-κB p65 (Abcam, Cambridge, UK) and secondary antibodies. The quantification was assessed using ImageJ. All used antibodies can be found in **Supplementary Table S5**, and full blots, including ponceau staining, are shown in **Supplementary Figures S2, S3**.

### 2.21 Proteomics

Incubated THP-1 cells were washed with PBS and lysed in 5% SDS in PBS. The cell lysate was incubated with benzonase HC, followed by incubation with 5 mM DTT and 40 mM CAA. The protein concentration was measured using a BCA assay kit (Thermo Fisher Scientific). The samples were frozen at −20°C until analyzed by our Proteomics Core facility. The data were analyzed using InstantClue.

### 2.22 Statistical analyses

GraphPad Prism (GraphPad Software, San Diego, CA, USA) was used for statistical analyses and graphs. Data are presented as scatter plots with mean + SEM. Normality was checked using normality and lognormality tests. Unpaired *t*-tests and Mann–Whitney *U* tests were performed for comparisons between the two groups. Kruskal–Wallis tests were used for comparisons between more than two groups. A *p*-value of < 0.05 was taken as statistically significant.

## 3 Results

### 3.1 MPO correlates with monocyte activity in HF patients

**Figure 1:**
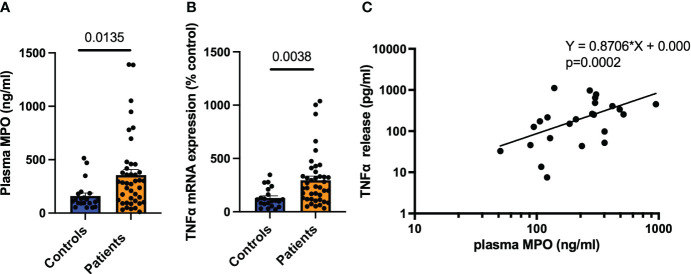
MPO correlates with monocyte activity in HF patients. MPO plasma levels of HF patients and healthy controls (*n* = 21/42) **(A)**. TNF-α mRNA expression of monocytes 2 h after isolation **(B)**. Correlation of TNF-α protein expression in monocytes versus MPO plasma levels **(C)**. Data are shown as mean + SEM. Unpaired *t*-tests and linear regression were performed for statistical analyses.

First, we investigated the connection between MPO and monocyte activation in HF. Patients show elevated MPO plasma levels (*p* = 0.0135) as well as higher TNF-α mRNA expression in isolated monocytes (*p* = 0.0038) compared to the age-matched healthy control group (**Figures 1A, B**). Interestingly, plasma MPO levels correlated to the TNF-α release of isolated monocytes across both groups (*p* = 0.0002, **Figure 1C**).

### 3.2 MPO deficiency alters splenic and cardiac leukocyte counts after MI

**Figure 2:**
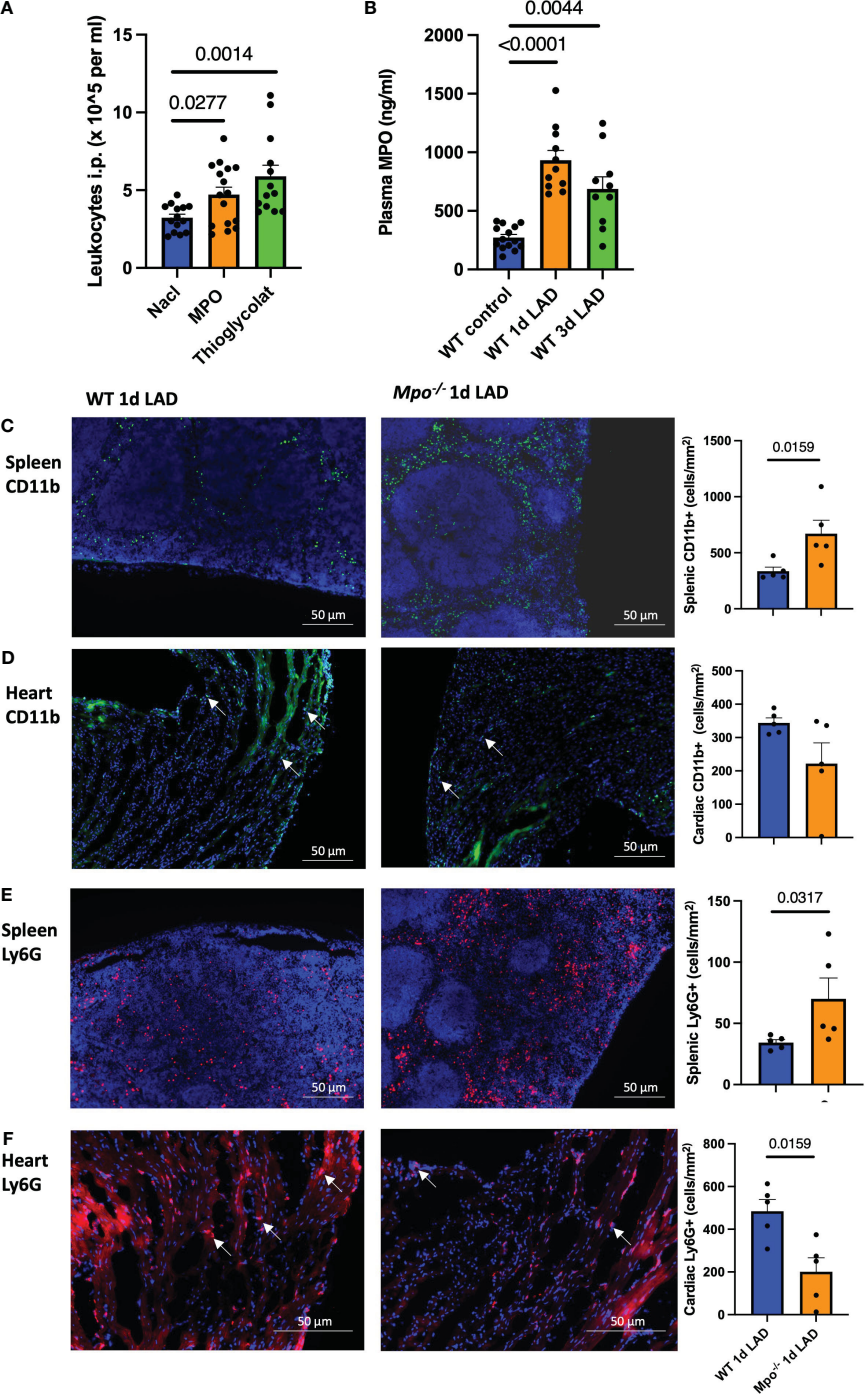
MPO induces splenic leukocyte recruitment into the heart after MI. Intraperitoneal leukocyte counts 3 days after MPO and thioglycolate i.p. injection **(A)**. Plasma MPO levels in WT mice at baseline and 1 day and 3 days after LAD ligation **(B)**. Representative immunofluorescence stainings and analyses of CD11b^+^ monocytes **(C, D)** and Ly6G^+^ PMN **(E, F)** in the spleen and left ventricle in WT and *Mpo^−/−^* mice 1 day after LAD ligation. Arrows indicate representative positive cells. Data are shown as mean + SEM. Kruskal–Wallis and Mann–Whitney *U* tests were performed for statistical analyses. WT, wildtype; *Mpo^−/−^*, MPO deficient; LAD, left anterior descending artery ligation.

To study the proinflammatory properties of MPO on leukocyte migration *in vivo*, *Mpo^−/−^* mice received an i.p. injection of either MPO, thioglycolate medium as positive control, or NaCl as a negative control. MPO and thioglycolate-injected mice showed markedly increased peritoneal infiltration of leukocytes compared to saline-injected control mice (*p* = 0.0277, *p* = 0.0014, respectively, **Figure 2A**). Additionally, mice showed increased MPO plasma levels after permanent LAD ligation, both at 1 day (*p* < 0.001) and 3 days (*p* = 0.0044) post-MI (**Figure 2B**). No plasma MPO was detected in *Mpo^−/−^* mice (data not shown). In accordance with the enhanced i.p. leukocyte infiltration after MPO injection, spleens of *Mpo^−/−^* mice showed a higher number of CD11b^+^ monocytes as demonstrated by immunofluorescence stainings compared to WT mice 1 day after LAD ligation, whereas cardiac monocyte infiltration was reduced (*p* = 0.0159, **Figure 2C**) (ns, **Figure 2D**). Similarly, *Mpo^−/−^* mice showed more Ly6G^+^ PMN in the spleen compared to WT mice (*p* = 0.0317, **Figure 2E**) but less in the heart (*p* = 0.0159, **Figure 2F**).

### 3.3 MPO enhances monocyte infiltration after MI

**Figure 3:**
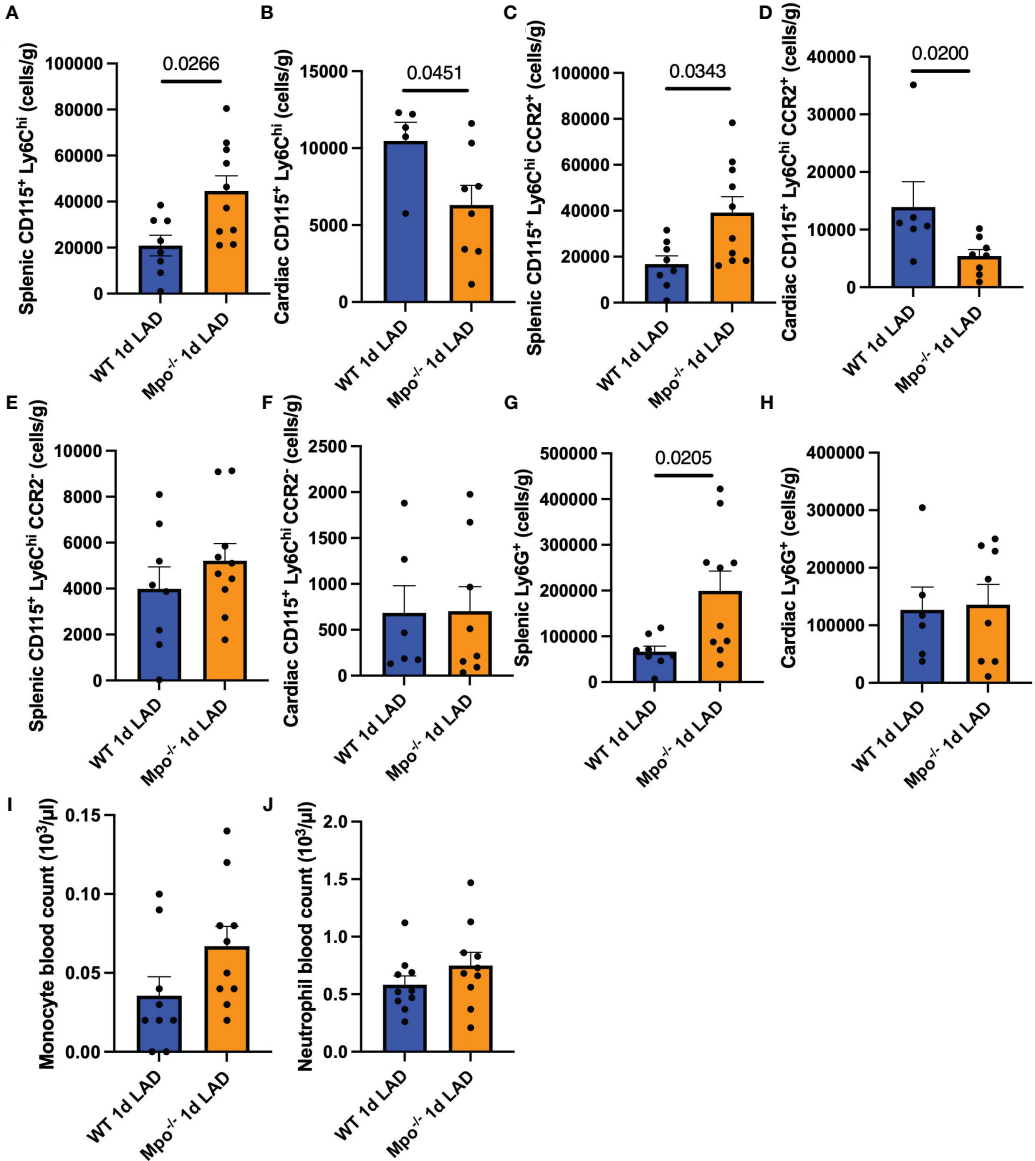
MPO enhances monocyte infiltration after MI. Flow cytometric analysis of splenic and cardiac CD115^+^Ly6C^hi^ monocytes **(A**, **B)**, splenic and cardiac CD115^+^Ly6C^hi^CCR2^+^ **(C**, **D)**, CD115^+^Ly6C^hi^CCR2^−^ monocytes **(E**, **F)**, splenic and cardiac Ly6G^+^ PMN **(G**, **H)**, and monocyte and PMN blood count **(I**, **J)** of WT and *Mpo^−/−^* mice 1 day after LAD ligation. Data are shown as mean + SEM. Mann–Whitney *U* tests were performed for statistical analyses. WT, wildtype; *Mpo^−/−^*, MPO deficient; LAD, left anterior descending artery ligation.

To characterize the effect of MPO splenic leukocyte migration into the myocardium after LAD ligation, spleen and heart tissues were stained with monocyte and PMN markers and analyzed by flow cytometry. The gating strategy is shown in **Supplementary Figure S1**. PMN were defined as live CD45^+^CD3e^−^Ly6G^+^ cells. Monocytes were defined as live CD45^+^CD3e^−^Ly6G^−^CD19^−^CD11b^+^CD115^+^Ly6C^hi^ cells. *Mpo^−/−^* mice showed elevated numbers of splenic CD115^+^Ly6C^hi^ monocytes (*p* = 0.0266) that were decreased in the left ventricular infarct region (*p* = 0.0451) as compared to WT mice (**Figures 3A, B**). Further analyses revealed that these monocytes were largely positive for the CCR2 receptor (**Figures 3C, D**). No difference in resident CD115^+^Ly6C^hi^CCR2^−^ monocyte numbers between both genotypes could be detected (**Figures 3E, F**). In accordance, *Mpo^−/−^* mice showed a higher number of Ly6G^+^ PMN in the spleen (*p* = 0.0205) as compared to WT controls (**Figures 3G, H**). Hematocytometric analyses showed no difference in monocyte and neutrophil blood count (**Figure 3I**).

### 3.4 MPO enhances monocyte migration via the upregulation of CCR2

**Figure 4:**
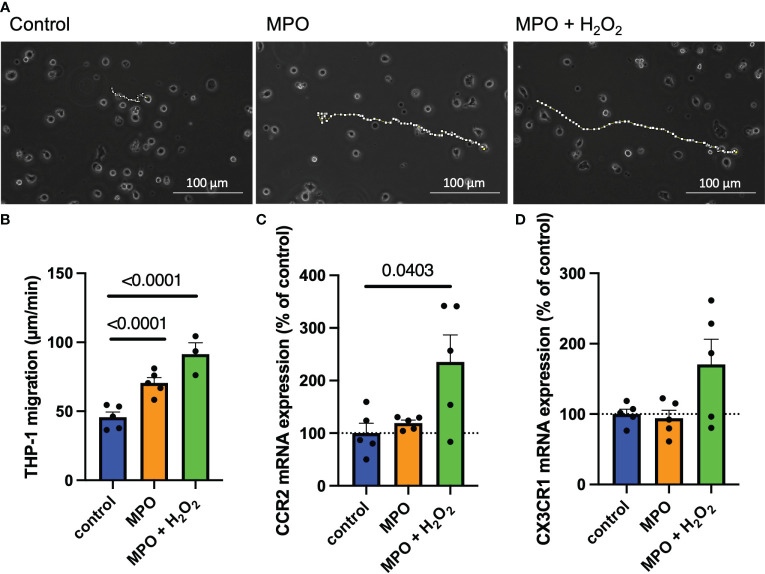
MPO enhances monocyte migration via upregulation of CCR2. Representative images and analyses of THP-1 cell migration after control-, MPO- or MPO + H_2_O_2_ treatment for 24 hours (dotted lines show the path of cell migration, each data point represents the mean of 10 tracked cells) **(A, B)**. CCR2 **(C)** and CX3CR1 **(D)** mRNA expression of THP-1 monocytes after control-, MPO- or MPO + H_2_O_2_ treatment for 24 hours. Data is shown as mean + SEM. Kruskal-Wallis tests were performed for statistical analyses.

To investigate if MPO enhances monocyte migration, THP-1 monocytes were exposed to MPO and MPO + H_2_O_2_ for 24 h. Their path of movement was tracked by live-cell imaging in an incubation chamber for 5 h (representative pictures are shown in **Figure 4A**). MPO treatment significantly increased THP-1 monocyte migration compared to control-treated cells (*p* < 0.0001). This effect was increased when cells were exposed to MPO + H_2_O_2_ (*p* < 0.001) (**Figure 4B**). Interestingly, mRNA expression analyses showed that MPO + H_2_O_2_, but not MPO alone, increased mRNA levels of the chemokine receptors CCR2 (*p* = 0.0403) and induced a nonsignificant increase in CX3CR1 mRNA expression (**Figures 4C, D**).

### 3.5 MPO increases NF-κB and p38 MAPK activation in THP-1 monocytes

**Figure 5:**
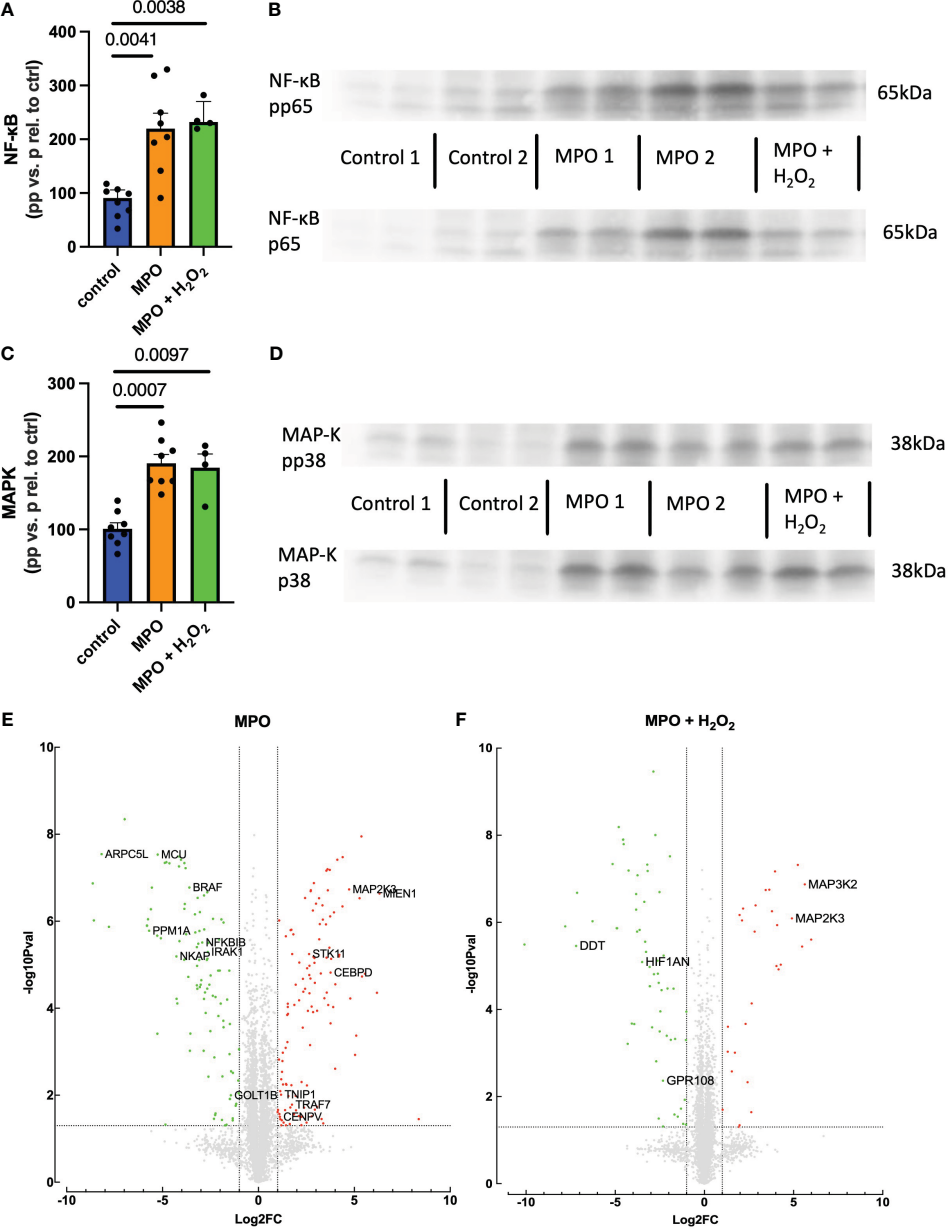
MPO increases NF-κB and p38 MAPK activation in THP-1 monocytes. NF-κB activation and example blots **(A**, **B)**. MAPK activation and example blots **(C**, **D)**. Volcano blots of proteomic analyses and a list of deregulated proteins **(E**, **F)**. Kruskal–Wallis and Student’s *t*-tests were performed for statistical analyses.

To examine the underlying mechanisms for increased monocyte migration, NF-κB and MAPK pathways were studied as the main inflammatory regulators known to impact CCR2 (29, 30). Phosphorylation of the p65 subunit of NF-κB and p38 MAPK was significantly enhanced in THP-1 monocytes when treated with MPO and MPO + H_2_O_2_ as compared to control-treated cells (**Figures 5A–D**). Proteomic analyses revealed how MPO-regulated genes are involved in migration and NF-κB and MAPK pathways (**Figures 5E, F**). NFKBIB, inhibiting NF-κB, and PPM1A, a negative regulator of MAPK, were downregulated in MPO-treated THP-1 monocytes compared to control (*p* < 0.001, *p* < 0.001, respectively). TNIP1 and MAP2K3, important in NF-κB and MAPK activation, respectively, were both upregulated in MPO-treated THP-1 monocytes (*p* = 0.01, *p* < 0.001, respectively). MAP2K3 was also upregulated in THP-1 monocytes treated with MPO + H_2_O_2_ compared to control (*p* < 0.001), as well as MAP3K2 (*p* < 0.001). Also, genes involved in migration were regulated by MPO. MIEN1, stimulating migration, was upregulated in the MPO-treated THP-1 monocytes compared to the control (*p* < 0.001), while DDT, inhibiting migration, was downregulated in THP-1 cells treated with MPO + H_2_O_2_ (*p* < 0.001). Other inflammatory mechanisms were affected as well, which are shown in **Figures 5E, F** and described in **Table 1**.

**Table 1:** Overview of proteins related to migration and inflammation deregulated by MPO or MPO + H_2_O_2_.

	Downregulated	Function	Upregulated	Function
**MPO**	ARPC5L	Cell migration	CENPV	Positive regulator of cytokinesis
	PPM1A	Negative regulator of MAPK	TNIP1	NFKB activation
	MCU	PMN chemotaxis	TRAF7	JNK activation
	NKAP	NFKB activation	CEBPD	Regulator of inflammatory responses
	BRAF	Regulator of MAPK/ERK	MAP2K3	MAPK activation
	NFKBIB	NFKB inhibitor	MIEN1	Cell migration
	IRAK1	IL-1 upregulation of NFKB		
	GOLT1B	Positive regulator of NFKB signaling		
**MPO + H_2_O_2_**	DDT	Migration inhibition	MAP2K3	MAPK activation
	HIF1AN	NFKB activation	MAP3K2	MAPK activation
	GPR108	Negative regulator TLR signaling		

## 4 Discussion

Myocardial infarction is followed by multifaceted inflammatory processes involving various immune cells and inflammatory mediators. Although inflammation after MI is crucial for cardiac healing, excessive inflammation induces adverse cardiac remodeling and subsequent HF. Neutrophils and monocytes arrive in the cardiac tissue within hours after the initial damage and play a crucial role in the early onset of the inflammatory phase. Neutrophils release high amounts of MPO, exerting immunomodulatory properties. Research has focused on MPO inhibition and controlling PMN infiltration into the infarcted tissue, but less is known about MPO’s role in monocyte migration and activation after MI. In this study, we identify MPO as a proinflammatory mediator of splenic monocyte recruitment and activation after MI via chemokine receptors such as CCR2 and activation of inflammatory pathways.

Increased plasma MPO levels are associated with cardiovascular disease (15–17). Accordingly, we showed increased plasma MPO in cardiac patients suffering from HF. Interestingly, the isolated monocytes of these patients showed increased inflammatory activation compared to the control group, as demonstrated by elevated TNF-α expression. Furthermore, we could reveal a correlation between the TNF-α release of isolated monocytes and plasma MPO levels. Although we acknowledge that our patient group not only included acute MI patients but also those with more chronic conditions, our findings sparked our interest in studying MPO’s role in splenic and cardiac monocyte activation and recruitment post-MI.

We demonstrated that MPO causes the migration of leukocytes into the peritoneum and continued to study MPO’s role in leukocyte migration in a mouse model of MI. MPO plasma levels were elevated and highest in the acute phase of inflammation 1 day after LAD ligation. Immunofluorescence staining of splenic tissue showed an increased PMN and myeloid cell count in *Mpo^−/−^* mice, while cardiac tissue showed lower PMN and (nonsignificant) lower myeloid cell numbers as compared to WT mice. Flow cytometric analyses showed comparable results, and identified the splenic and cardiac myeloid cells as CD115^+^Ly6C^high^ monocytes. These monocytes were largely CCR2^+^, suggesting a role for MPO in monocyte migration.

Monocytes and neutrophils are the first immune cells to arrive in the injured tissue, both infiltrating within the first day after an infarction. This is also confirmed by our immunofluorescence and flow cytometry data. Both cell types secrete cytokines, chemokines, and growth factors, which induce further migration, and both cell types contain MPO, though MPO concentrations in PMN are higher. MPO has already been proven to attract PMN by glycocalyx interaction via its cationic charge (31), inducing PMN migration to the site of infarction.

Live-cell imaging using the human monocytic cell line THP-1 showed that MPO induces migration of monocytes. This is, at least in part, dependent on increased expression of the chemokine receptors CCR2 and CX3CR1, with the highest effect related to MPO’s catalytic function in the presence of its principal substrate, H_2_O_2_. This shows that MPO not only induces PMN migration by physical forces (31) but is also involved in monocyte migration via upregulation of chemokine receptors.

Mechanistically, we looked into MAPK and NF-κB pathways, as these are known inflammatory mediators and impact CCR2 activation and secretion (29, 30). THP-1 monocytes showed increased MAPK and NF-κB protein activation when treated with MPO or MPO and H_2_O_2_, either due to an increase in phosphorylation or an increase in total protein levels. Our proteomic analyses further demonstrated that MPO regulates genes involved in migration and the NF-κB and MAPK pathways. NFKBIB, inhibiting NF-κB, and PPM1A, a negative regulator of MAPK, are downregulated by MPO. TNIP1 and MAP2K3, important in NF-kB and MAPK activation, respectively, are both upregulated in MPO-treated THP-1 monocytes. MAP2K3 was also upregulated in THP-1 monocytes treated with MPO + H_2_O_2_ compared to control, as well as MAP3K2, further supporting a role for MPO in MAPK activation. MPO also regulated genes involved in migration. MIEN1, stimulating migration, is upregulated in the MPO-treated THP-1 monocytes compared to control, while DDT, inhibiting migration, is downregulated in THP-1 cells treated with MPO + H_2_O_2_.

MAPK and NF-κB both play a role in cardiac healing post-MI. HOCl, an MPO-derived oxidant, activates MAPK (32), which in turn stimulates fibroblast-to-myofibroblast transdifferentiation (17), thereby increasing collagen production and fibrosis after MI (33) and hence promoting maladaptive remodeling. NF-κB, a key mediator in inflammation, promotes cytokine and chemokine production and, hence, cell migration toward the site of injury. The role of MPO and its products on NF-κB activation is controversial and depends on concentration and cell type. MPO directly showed activation of PMN by binding to the CD11b receptor, while HOCl triggered inhibition of NF-κB and subsequent CCR2 expression in keratinocytes and melanoma cells (34). Hypothiocyanous acid (HOSCN), another oxidative product of MPO’s enzymatic activity, is also associated with NF-κB activation in macrophages (35). In a leukocyte context, MPO is generally regarded as proinflammatory; however, it is noteworthy that higher concentrations of HOCl have been associated with leukocyte cell death as well (36).

MI causes leukocytosis and a massive infiltration of monocytes into the injured heart (37). Monocytes in the infarct have a turnover of less than 24 h (38), meaning the high demand must be met by hematopoietic organs such as bone marrow and the spleen. The majority of immune cells derive from hematopoietic stem and progenitor cells (HSPCs) via hematopoiesis in the bone marrow, but extramedullary sites such as the spleen and liver contribute when the demand is high, especially in the acute phase of inflammation (39, 40). Indeed, splenic myelopoiesis replenishes the cardiac monocyte reservoir, supplying cells to the infarcted tissue during the first 24 h after MI in a CCR2-dependent manner (13). While we showed increased CCR2^+^ splenic monocyte count in *Mpo^−/−^* mice compared to WT and yet less CCR2^+^ monocytes in the infarcted heart, we hypothesize that MPO is involved in the recruitment of splenic monocytes toward the infarcted heart and could be involved in myelopoiesis too. However, we did not include bone marrow and other extramedullary sites in our work, which is subject to future studies.

Therapeutic inhibition of MPO is proven to have protective effects on cardiac function in murine models of MI via reduced infiltration of neutrophils and Ly6C^high^ monocytes (17, 24, 25). Currently, a specific MPO inhibitor, AZD4831, is subject to (pre)clinical studies (26, 27). The importance of advancing anti-inflammatory therapies following MI is underscored by clinical trials. The CANTOS trial demonstrated that anti-inflammatory therapy targeting the IL-1ß pathway with canakinumab lowered recurrent cardiovascular events in patients with previous MI and elevated CRP levels (41). In another clinical trial, the LoDoCo trial, a low dose of the anti-inflammatory drug colchicine reduced the risk of cardiovascular events in patients with chronic coronary disease (42, 43). We identified MPO-induced CCR2 expression as a driving mechanism for increased cardiac monocyte infiltration post-MI. We therefore propose that inhibiting MPO may offer an anti-inflammatory therapeutic approach, reducing CCR2-dependent migration and reducing MAPK and NF-κB activation. This study establishes a connection between MPO and CCR2-induced monocyte migration, which opens up an exciting new therapeutic avenue. For instance, the possibility of a dual therapy involving MPO and CCR2 inhibition could be considered an anti-inflammatory treatment for ischemic myocardial disease. Given the existence of clinical CCR2 inhibitors, this provides, besides pharmacological MPO inhibition, two potential new avenues for anti-inflammatory therapies for ischemic HF patients.

Our work has some limitations. Murine models can only partly resemble the human physiology and cardiovascular system. Furthermore, this study primarily focuses on the acute phase, 1 day after MI, in which the spleen serves as the main recruitment site for cardiac-infiltrating monocytes (13). In future studies, it would be of interest to investigate the impact of MPO on bone marrow monocyte recruitment and myelopoiesis. Additionally, we have mainly focused on circulating MPO rather than the effects of intracellular MPO on monocyte migration and activation. However, monocyte-derived MPO has a relatively low impact on cardiovascular diseases (44) and likely does not play a significant role in the mechanisms under investigation here. Next, although our results suggest that MPO primarily stimulates the recruitment of monocytes into the heart after MI through the expression of CCR2, we cannot completely exclude the possibility that MPO also influences monocyte retention within the spleen. Specifically, MPO was found to promote the adhesion of leukocytes to the endothelium (31). This aspect of MPO-induced monocyte recruitment still requires further investigation, as does the interaction of activated monocytes with endothelial cells, given the known effects of MPO on the endothelium (45, 46).

## 5 Summary

Inflammatory processes are essential for proper tissue repair after MI, though excessive inflammation caused by MPO results in adverse cardiac remodeling. MPO inhibition showed protective effects on cardiac function after myocardial infarction and is currently subject to (pre)clinical studies. The underlying mechanisms are, however, largely unknown. Here, we postulate that MPO not only stimulates neutrophil migration but also induces the migration of splenic monocytes toward the ischemic heart. This is due to the monocytic upregulation of chemokine receptors such as CCR2 and the activation of inflammatory pathways, e.g., NF-κB and MAPK. Taken together, this study reveals for the first time a connection between MPO and CCR2-induced migration, suggesting potential applicability also in other pathologies involving PMN and CCR2, and shows that MPO plays an important role in inflammation after MI, providing mechanistic insight for novel therapeutic strategies.

## Data availability statement

The original contributions presented in the study are included in the article/**Supplementary Materials**. Further inquiries can be directed to the corresponding author.

## Ethics statement

The studies involving humans were approved by ethics application number 13-019. The studies were conducted in accordance with the local legislation and institutional requirements. The participants provided their written informed consent to participate in this study. The animal study was approved by State Agency for Nature, Environment and Consumer Protection (LANUV), Recklinghausen, NRW, Germany and under license numbers 2014.A234 and 2020.A487. The study was conducted in accordance with the local legislation and institutional requirements.

## Author contributions

VP: Conceptualization, Formal Analysis, Investigation, Methodology, Visualization, Writing – original draft, Writing – review & editing. FM: Formal Analysis, Investigation, Methodology, Visualization, Writing – review & editing. IE: Formal Analysis, Investigation, Methodology, Visualization, Writing – review & editing. HN: Investigation, Methodology, Writing – review & editing. DMu: Investigation, Methodology, Writing – review & editing. MTou: Investigation, Methodology, Writing – review & editing. MTor: Investigation, Methodology, Writing – review & editing. DMe: Methodology, Writing – review & editing. SG: Methodology, Writing – review & editing. FN: Methodology, Writing – review & editing. FP: Methodology, Writing – review & editing. HG: Methodology, Writing – review & editing. AH: Methodology, Writing – review & editing. PA: Methodology, Writing – review & editing. BA: Investigation, Writing – review & editing. AK: Methodology, Writing – review & editing. VR: Methodology, Writing – review & editing. HH: Methodology, Writing – review & editing. DB: Methodology, Writing – review & editing. MA: Methodology, Writing – review & editing. FH: Methodology, Writing – review & editing. HW: Methodology, Writing – review & editing. SB: Methodology, Writing – review & editing. MM: Conceptualization, Methodology, Supervision, Writing – review & editing.
