# Supplementary material for: Myeloperoxidase induces monocyte migration and activation after acute myocardial infarction
Source: Front Immunol. 2024 Apr 26;15:1360700. doi: 10.3389/fimmu.2024.1360700 (PMC11082299; doi:10.3389/fimmu.2024.1360700)
Supplement: Supplementary file 1 [file DataSheet_1.pdf]

Supplement

		Patients	*Controls*	
		n = 42	n = 21	p-value
Age		66,2 ± 13,3	63,3 ± 9,8	0,30
Sex,	female	10 (23,8)	10 (47,6)	0,09
	male	32 (76,2)	11 (52,4)	
LVEF, %		30,7 ± 9,4	/	
Ethiology	ICM	25 (59,5)	/	
	DCM	11 (26,2)	/	
	Hypertrophy	1 (2,4)	/	
	Myocarditis	2 (4,8)	/	
	Unknown	3 (7,2)	/	
Hypertension		23 (54,8)	11 (52,4)	1,00
Hypercholesterolemia		22 (52,4)	8 (38,1)	0,42
Diabetes		11 (26,2)	2 (9,5)	0,19
Smoking	current	6 (14,3)	3 (14,3)	0,53
	former (>10 years)	7 (16,7)	6 (28,6)	
rel. Kidney failure	(>CKD3)	16 (38,1)	0 (0,0)	<0,01

Supplementary Table 1 Group characteristics. Values presented as n (%) or mean ± SD; significance tested by Fischer's exact test or Mann-Whitney U test; LVEF, left ventricular ejection fraction; ICM, ischemic cardiomyopathy; DCM, dilated cardiomyopathy; CKD, chronic kidney disease staging.

Target	FWD	REV
GADPH, human	5’ aatcccatcaccatcttcca 3’	5’ ttcacacccatgacgaacat 3’
TNFa, human	5‘ gctgcactttggagtgatcgg 3‘	5‘ gaggtacaggccctctgatg 3‘
CCR2, human	5‘ gtgtgtggaggtccaggagt 3‘	5‘ caacccagctggagtctctc 3‘
CX3CR1, human	5‘ gacggttgcatttagccatt 3‘	5‘ tgctcagaacacttccatgc 3‘
18s, human	5‘ ggggcaagatcctcactttc 3‘	5‘ ctctggcacgctcgaact 3‘

Supplementary Table 2. Used primer sequences for mRNA analyses of isolated human monocytes, and stimulated THP-1 monocytes.

Target	Dilution	Host	Direct/2nd	nm	Source
Fc-Block Anti-mouse CD16/CD32	1:100	Rat	-	-	BD
Ly6G	1:200	Rat IgG2a	Direct	594 red	BioLegend
Ly6G Isotype control	1:200	Rat IgG2a	Isotype control	594 red	BioLegend
Anti-mouse CD11b	1:200	Rat IgG2b	Direct	488	Invitrogen
Isotype control CD11b	1:200	Rat IgG2b	Isotype control	488	Invitrogen

Supplementary Table 3. Used antibodies for immunofluorescent staining of neutrophils and myeloid cells in cardiac and splenic tissue.

Fluor	Target	Clone	Dilution	Source
BUV496	CD45	30-F11	1:200	BD
BUV563	Ly6G	1-A8	1:200	BD
BUV661	CD11b	M1/70	1:400	BD
BUV737	CD3e	145-2C11	1:150	BD
BV650	Ly6C	HK1.4	1:100	BioLegend
BV785	CCR2	SA203G11	1:100	BioLegend
Spark NIR 685	CD19	6D5	1:400	BioLegend
AF488	CD115	AFS98	1:100	BioLegend

Supplementary Table 4. Used antibodies for flow cytometric analyses of neutrophils and monocytes in cardiac and splenic tissue.

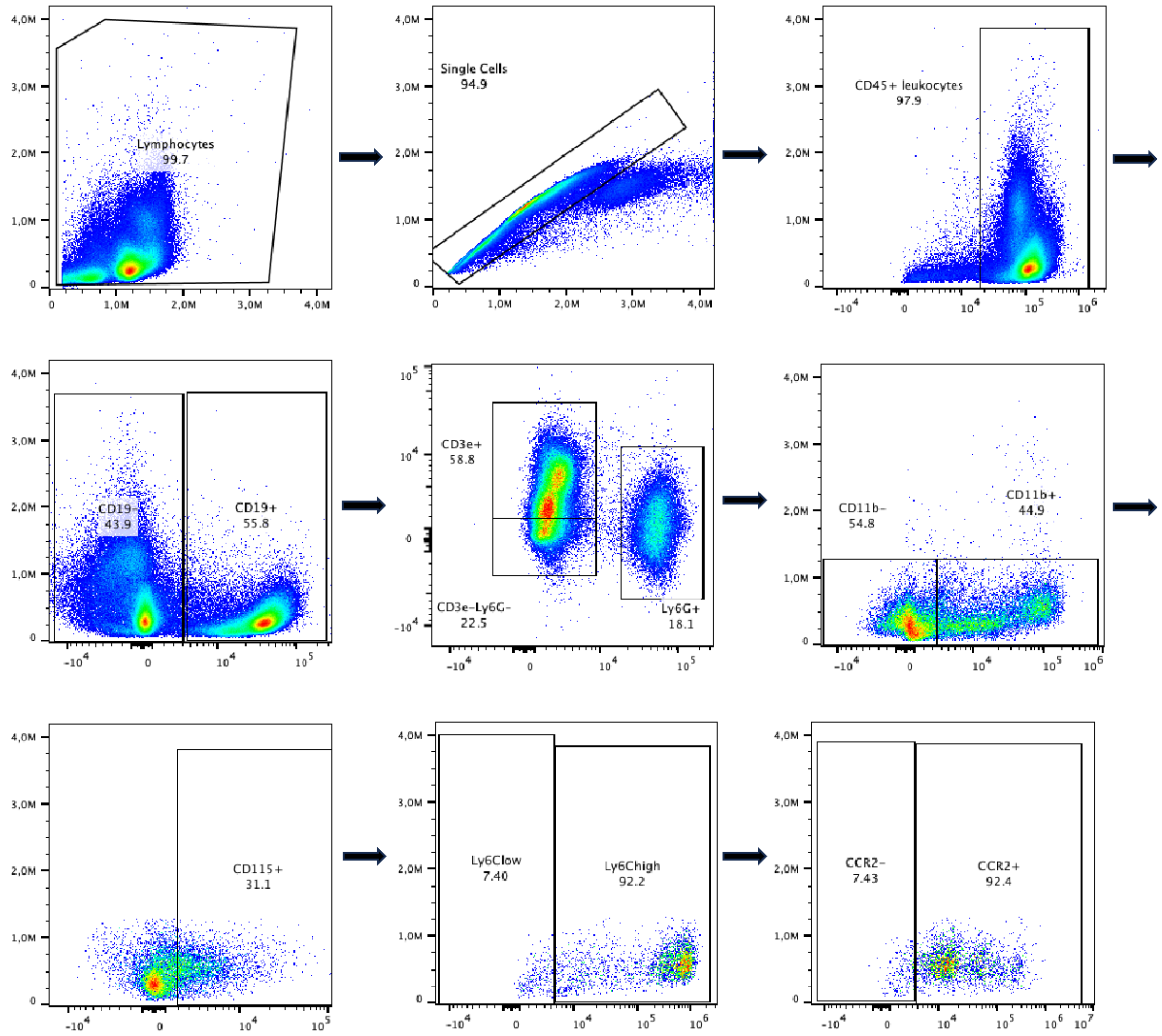

Supplementary Figure 1. Gating strategy for defining neutrophils and monocytes in cardiac and splenic tissue. Neutrophils were defined as live CD45$^+$CD3e$^-$Ly6G$^+$ cells. Monocytes were defined as live CD45$^+$CD3e$^-$Ly6G$^-$CD19$^-$CD11b$^+$CD115$^+$Ly6C^{hi} cells.

Target	Dilution	Host	Source
MAP-K p38	1:1000	Mouse	Thermofisher
pp38 MAP-K	1:1000	Rabbit, polyclonal	Cell signaling
pp65 NF-kappa B	1:1000	Rabbit, monoclonal	Invitrogen
p65 NF-kappa B	1:1000	Rabbit Polyclonal	Abcam
IgG (H+L), peroxidase		Horse	Vector Laboratories
IgG (H+L), peroxidase		Goat	Vector Laboratories

Supplementary Table 5. Used antibodies for western blotting of THP-1 monocytes.

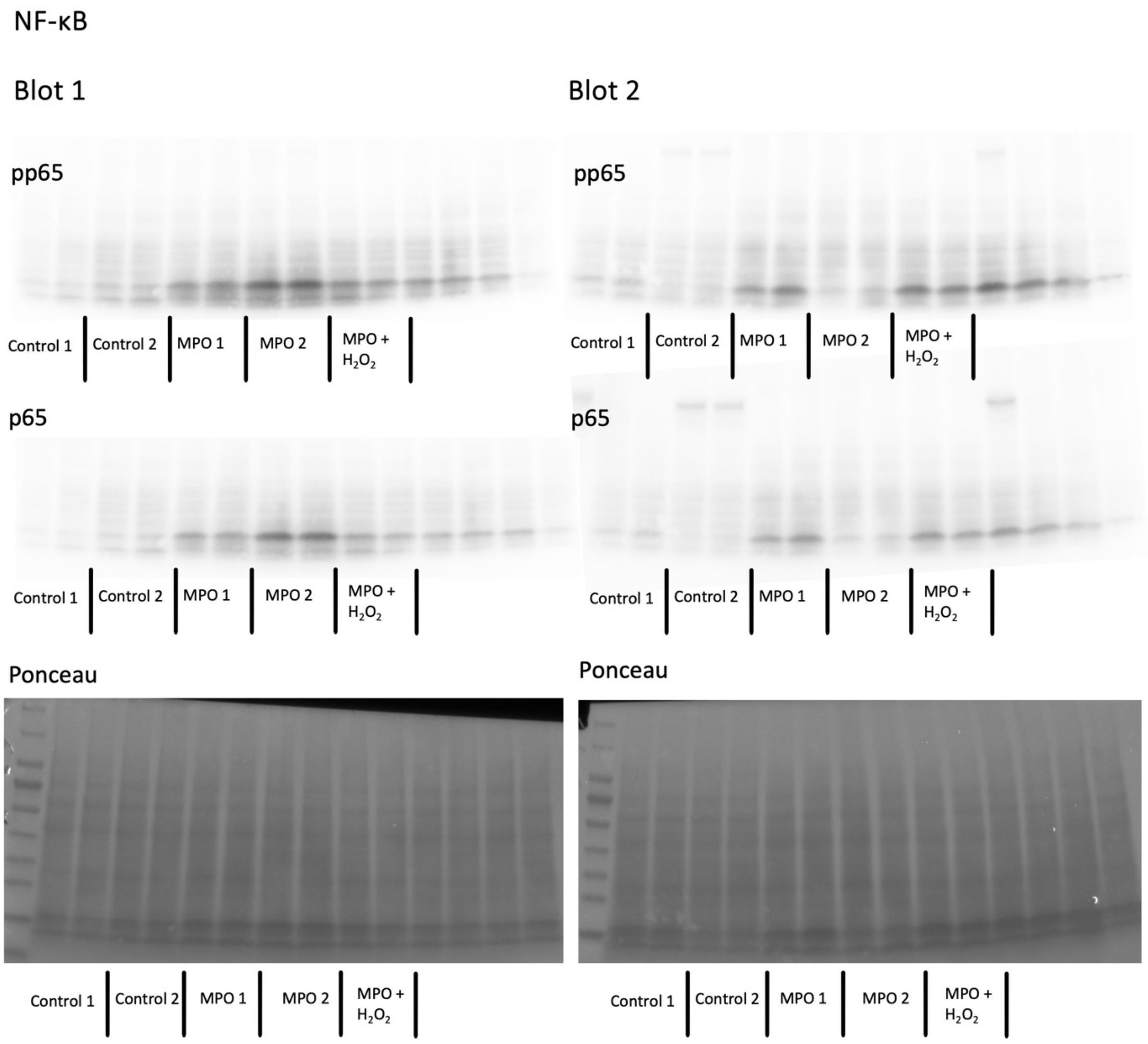

Supplementary Figure 2. NF-κB blots for pp65 and p65, and ponceau staining.

MAPK

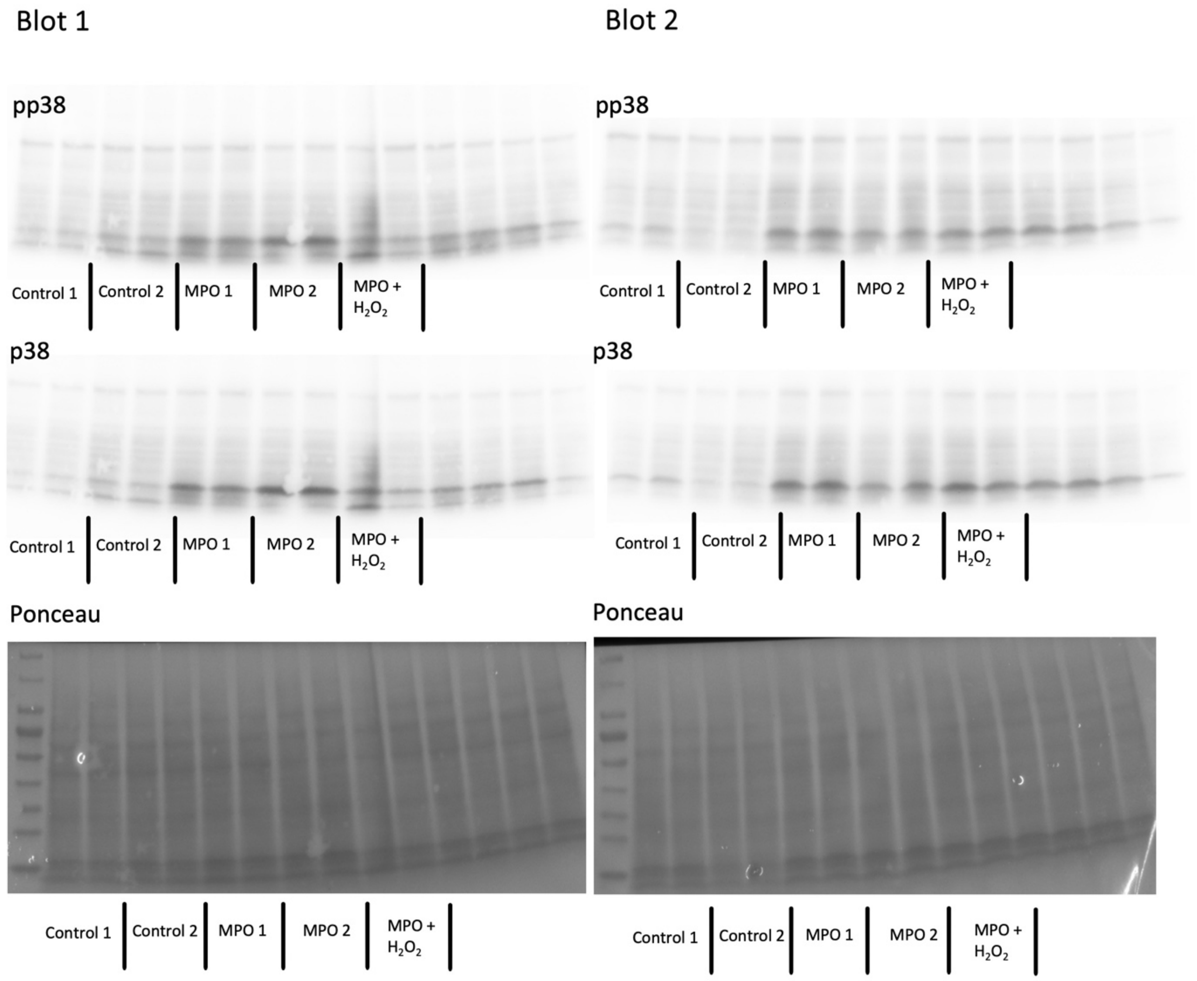

Supplementary Figure 3. MAPK blots for pp38 and p38, and ponceau staining.
